# Hydrophilic Modification of Multi-Walled Carbon Nanotube for Building Photonic Crystals with Enhanced Color Visibility and Mechanical Strength

**DOI:** 10.3390/molecules21050547

**Published:** 2016-04-28

**Authors:** Feihu Li, Bingtao Tang, Jinghai Xiu, Shufen Zhang

**Affiliations:** State Key Laboratory of Fine Chemicals, Dalian University of Technology, Dalian 116024, China; lifeihu@mail.dlut.edu.cn (F.L.); xiujh123@126.com (J.X.); zhangshf@dlut.edu.cn (S.Z.)

**Keywords:** carbon nanotube, hydrophilic modification, structural color, color visibility, mechanical strength

## Abstract

Low color visibility and poor mechanical strength of polystyrene (PS) photonic crystal films have been the main shortcomings for the potential applications in paints or displays. This paper presents a simple method to fabricate PS/MWCNTs (multi-walled carbon nanotubes) composite photonic crystal films with enhanced color visibility and mechanical strength. First, MWCNTs was modified through radical addition reaction by aniline 2,5-double sulfonic acid diazonium salt to generate hydrophilic surface and good water dispersity. Then the MWCNTs dispersion was blended with PS emulsion to form homogeneous PS/MWCNTs emulsion mixtures and fabricate composite films through thermal-assisted method. The obtained films exhibit high color visibility under natural light and improved mechanical strength owing to the light-adsorption property and crosslinking effect of MWCNTs. The utilization of MWCNTs in improving the properties of photonic crystals is significant for various applications, such as in paints and displays.

## 1. Introduction

Carbon nanotubes (CNTs) have been intensively investigated over the past years because of their unique properties and widespread applications [1,2,3,4,5,6]. For example, CNTs are applied in solar thermal energy storage owing to their excellent light absorption ability [7,8]. CNTs are considered ideal candidates for polymer reinforcement and for fabricating ultra-strong composite materials owing to their excellent mechanical properties in the direction of the tubule axis [9,10]. However, the major drawback of CNTs is their inherent insolubility in most organic and aqueous solvents and the strong tendency to “rope up” in solutions, which seriously hinder their molecular level processing and further practical applications [11,12]. Considerable efforts have been exerted to prepare their stable, homogeneous, and aggregation-free dispersions. Functionalization or modification of carbon-based materials is an effective approach, which can be classified into three categories: (a) covalent attachment of chemical groups through reactions onto the π-conjugated skeleton; (b) noncovalent adsorption or wrapping of various functional molecules; and (c) endohedral filling of the inner empty cavity [1,12,13,14,15,16]. The use of diazonium chemistry for the functionalization of carbon materials is convenient because of its unique advantage. The modified surface property can be adjusted by choosing specific functional groups of the grafted aryl moieties that suitable for desired applications [15,17,18,19].

Recently, the utilization of black materials as additive has been gained interest in fabricating photonic crystals with enhanced optical properties [20,21]. General photonic crystal made from simple PS, polymethylmethacrylate (PMMA) or silicon dioxide (SiO_2_) exhibit iridescence structural colors with a low color visibility, which limits their potential applications. Black materials were set as absorbing agent of scattered light in common studies to get enhanced color. For example, carbon black (CB) and black dye have been used as doping agent to obtain photonic crystal films with enhanced structural color visibility [22,23,24,25,26,27]. Moreover, general photonic crystals, such as PS films, also suffer from poor mechanical strength because of weak interaction among colloidal spheres. As a special kind of carbon material, CNTs possess excellent light absorption performance and mechanical properties. Thus, utilization of CNT in the fabrication of photonic crystals may provide immediate enhancement both in the optical and mechanical properties.

In the present study, crude MWCNTs were successfully modified through radical addition reaction via aniline 2,5-double sulfonic acid diazonium salt. The modified MWCNTs have hydrophilic surface and good water dispersity, which are suitable for solution processing. Then, the MWCNTs dispersion was blended with PS emulsion to fabricate photonic crystals. The structural color visibility of the obtained PS/MWCNTs composite films under natural light were greatly enhanced because of the light-absorbing property of the doped MWCNTs. In addition, the mechanical properties of the PS films were also improved because of the sufficient interaction and crosslinking effect among PS colloidal particles and MWCNTs.

## 2. Results and Discussion

### 2.1. Dispersion Behavior of Modified MWCNTs

The major disadvantage of CNTs is their insolubility and strong tendency to “rope up” in solutions, which hamper molecular level studies and applications. Thus, surface modification was employed to obtain hydrophilic MWCNTs and improve their dispersibility in water. Dispersion behavior testing was carried out to confirm the effect of modification. As shown in Figure S1, the crude MWCNTs had an extremely poor wettability and only float on the water surface (Figure S1a). However, the modified MWCNTs can be easily dispersed in water, forming homogeneous dispersion even after centrifugation (Figure S1b). The UV-Vis spectra of the upper, middle, and bottom of the MWCNTs dispersion were measured and the corresponding spectra of three different positions nearly coincide with each other, as can be seen in Figure S2. This further demonstrates that the modified MWCNTs have sufficient surface hydrophilicity and can be easily dispersed in water to form homogeneous dispersion. In addition, homogeneous PS/MWCNTs emulsion mixtures can be easily obtained after blending the modified MWCNTs with PS emulsion (Figure S1c).These clearly illustrate successful modification and good dispersion behavior.

### 2.2. Characterization of the Modified MWCNTs

**Figure 1:**
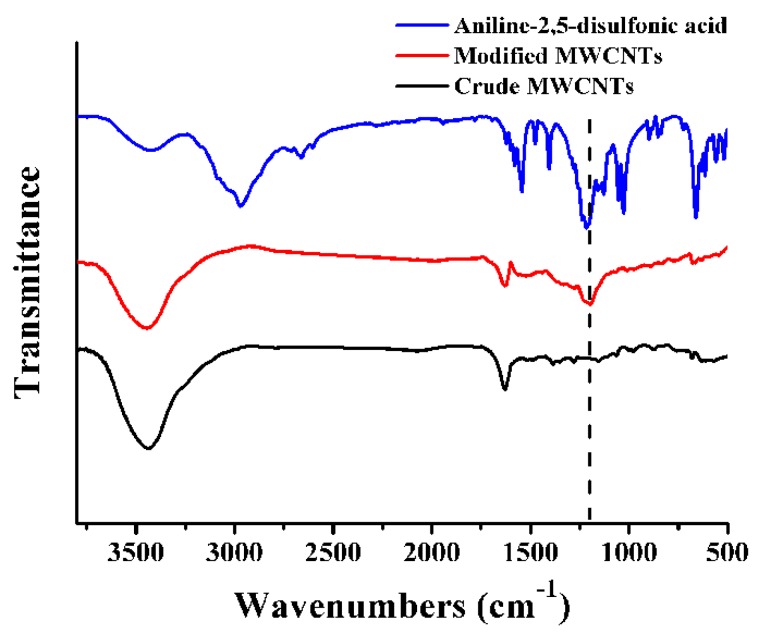
Fourier transform infrared (FT-IR) spectra of the crude and modified multi-walled carbon nanotubes (MWCNTs).

Fourier transform infrared (FT-IR) spectroscopy was employed to verify the successful modification MWCNTs. As shown in Figure 1, the strong absorption peaks in the spectra of both the crude and modified MWCNTs at 1631 cm^−1^ are attributed to their aromatic C=C bond vibration. Importantly, the strong absorption peak at 1197 cm^−1^, which belongs to the characteristic S=O bond vibrations of sulfonic acid groups, both appeared in the FT-IR spectra of the modified MWCNTs and aniline 2,5-double sulfonic acid. XPS analysis was carried out to identify the change of elemental composition of modified MWCNTs. New peak which belongs to S_2p_ core appeared at 168 eV in the XPS spectrum of modified MWCNTs compared to that of crude MWCNTs (Figure S3). TGA analysis was also carried out to measure weight fraction of the organic portion bonded to the modified MWCNTs. As can be seen in Figure S4, crude MWCNTs have no weight loss in the whole range of testing temperature. However, the modified MWCNTs have a weight loss of about 8% in the range of 200–600 °C. Thus, hydrophilic group which contain sulfonate group were successfully grafted onto the surface of MWCNTs. Zeta potential is an important characteristic that expresses the stability of colloidal dispersions. After modification, the zeta potential of MWCNTs dispersions was −56 mV, thereby guaranteeing the good stability of the dispersions. These results demonstrated the ideal result of the modification.

### 2.3. Effect of MWCNTs on the Color of Photonic Crystal Films

**Figure 2:**
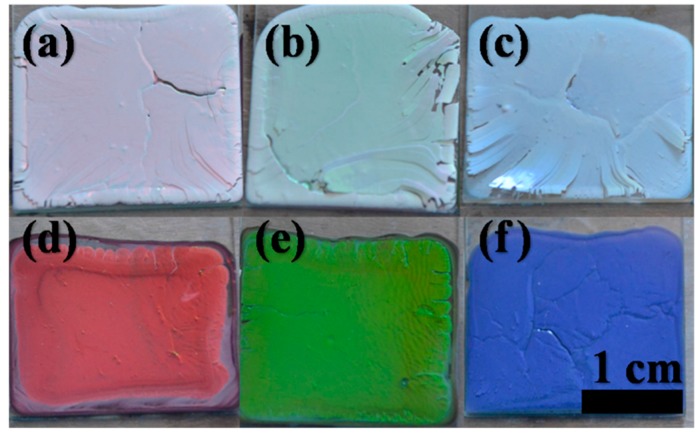
Optical photographs of the prepared structural color films with and without MWCNTs indoors under natural light without an external light source. (**a**), (**b**) and (**c**): red, green and blue films without MWCNTSs; (**d**), (**e**) and (**f**): corresponding films doped with MWCNTs; Hydrodynamic particle sizes were 317, 261, and 235 nm, respectively.

Photonic crystal films can exhibit inherently structural colors because of the Bragg reflection of the periodic arrangement of colloidal spheres with a specific particle sizes [28,29,30]. However, these particles (such as PS) exhibit faint structural colors because of light scattering, which produces milky white colors and a low color visibility under natural light [20,31]. In this study, black MWCNTs were used as additive to fabricate photonic crystals. Homogeneous PS/MWCNTs emulsion mixture can be easily obtained after blending with modified MWCNTs. Then, PS and PS/MWCNTs emulsions were self-assembled into structural color films through thermal-assisted method. Three PS emulsions with hydrodynamic particle sizes of 317, 261, and 235 nm were used. Figure 2 shows the optical images of the self-assembled films with and without MWCNTs on the glass substrate under natural light. As shown in Figure 2a–c, films with three structural color of red, green, and blue were generated from the assembly of the PS emulsion without MWCNTs. However, these structural colors suffer from low visibility and saturability under natural light without an external light source. When 0.1 wt % of MWCNTs was doped, the color visibility and brightness was strongly enhanced (Figure 2d–f).

**Figure 3:**
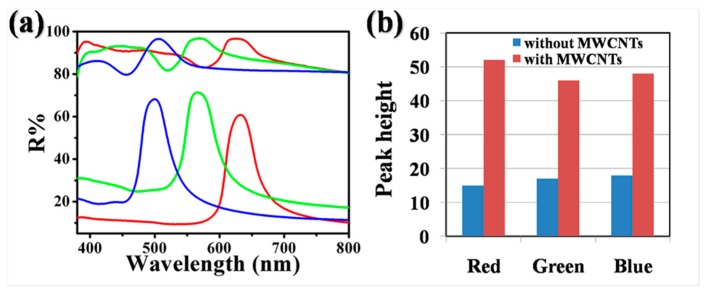
(**a**) Reflection spectra of the corresponding red, green and blue structural color films without MWCNTs (upper regions) and with MWCNTs (bottom regions); (**b**) The reflection peak intensity contrast of the structural color films without and with MWCNTs.

This result can be explained by the strong absorption characteristic of black MWCNTs in the visible light region, thereby effectively absorbing the scattering light and background light [22,23,24,32]. Figure 3a shows the corresponding reflection spectra of the structural color films, which further demonstrated the effect of black MWCNTs on the structural colors. When MWCNTs were added, the peak intensity was significantly enhanced compared with undoped PS films. The reflection peak intensity contrast in Figure 3b provides a more distinct expression of this change. Thus, the color visibility was enhanced because the structural color brightness was dependent on the reflectance intensity between the stop band and the wavelengths outside the stop band.

**Figure 4:**
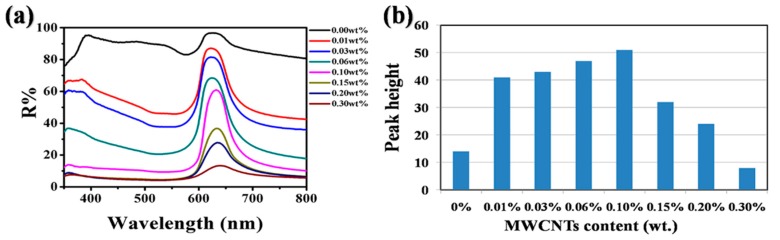
(**a**) Reflectance spectra of the PS/MWCNTs films with different MWCNTs content (PS particle size was 317 nm); (**b**) Corresponding reflection peak intensity contrast.

Furthermore, the effect of the doping amount of MWCNTs on the optical properties of the PS/MWCNTs composite films was investigated by reflectance spectroscopy. Different amount of MWCNTs were mixed with PS emulsion to fabricate PS/MWCNTs composite photonic crystal films. PS emulsion with hydrodynamic particle size of 317 nm was selected as representative sample. The result shown that the color visibility and brightness were enhanced gradually with the increase of MWCNTs content. The reflection peak intensity (*i.e.*, the peak amplitude) contrast of different sample further explain this phenomenon. As can be seen in Figure 4a,b, reflection peak intensity increased with the increase of MWCNTs content, but when the MWCNTs content was more than 0.1 wt %, the peak intensity started to decrease owing to the serious absorption of the whole region of light.

### 2.4. Effect of MWCNTs on the Mechanical Strength of Photonic Crystal Films

**Figure 5:**
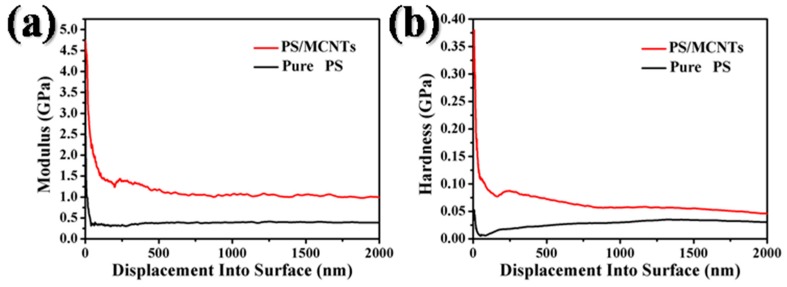
(**a**) Curves of modulus for the PS/MWCNTs and pure polystyrene (PS) films as a function of displacement into surface (nm); (**b**) Curves of hardness for the PS/MWCNTs and pure PS films as a function of displacement into surface (nm); The test sample was the red film.

**Figure 6:**
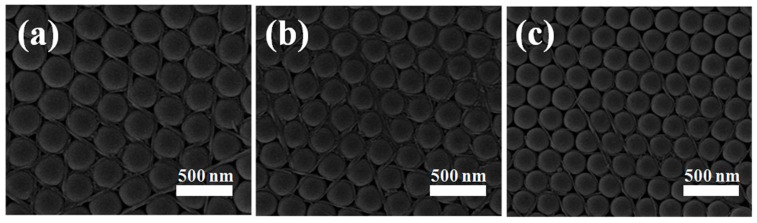
SEM images showing the cross-linking effect between MWCNTs and PS particles: (**a**) red film; (**b**) green film; (**c**) blue film.

The self-assembly of monodisperse colloidal spheres into ordered array structure provides a simple and convenient approach to prepare photonic crystals. However, colloidal crystals suffer from very low mechanical strength because of weak interaction among colloidal spheres. As shown above in Figure 2a–c, the obtained pure PS film was fragile and exhibited apparent cracks on their surface. In contrast, the PS/MWCNTs composite films have a flat surface and the cracks disappear completely when 0.1 wt % of MWCNTs was doped (Figure 2d–f). To evaluate the difference of their mechanical strength, modulus and hardness were measured by Nanoindentation. The curves in Figure 5a,b show that the hardness and modulus of PS/MWCNTs film were enhanced significantly compare with that of pure PS films, which indicated the improvement of the mechanical strength. These phenomena can be explained by the sufficient contact and interaction between MWCNTs and PS particles owing to the high aspect ratio (surface/area ratio) and considerable longitudinal length of MWCNTs. Namely, MWCNTs acted as “crosslinkers”, and the crosslinking effect facilitated the improvement of mechanical strength, which is confirmed by the SEM images. As shown in Figure 6 (0.1 wt % MWCNTs doping content), MWCNTs filled in the gaps of PS colloidal array, which formed a network, thereby effectively linking the particles. Figure S5 shows the SEM image of red pure PS film.

## 3. Materials and Methods

### 3.1. Materials

MWCNTs with 8–15 nm diameter and 0.5–2 μm length (specific surface area: >233 m^2^/g, purity: >95%) were purchased from China Sciences Academy Chengdu Organic Chemical Co., Ltd. (Chengdu, China). Aniline-2,5-disulfonic acid monosodium salt, styrene (St), sodium dodecyl sulfate (SDS) and sodium nitrite were purchased from DAMAO Chemical Reagent Factory (Tianjin, China). Anhydrous sodium carbonate and potassium peroxydisulfate (KPS) were purchased from BODI chemical Industry Co., Ltd. (Tianjin, China). Deionized water was used in all the experiments. All chemical reagents were used without further purification.

### 3.2. Characterization

The average hydrodynamic sizes of the particles were measured by particle size analyzer (DLS) (Zetasizer 1000, Malvern, Malvern City, UK). Optical photographs of PS photonic crystal films were taken with a Nikon D7000 digital camera. The microstructures of films were observed with scanning electron microscopy (SEM, NOVA NANOSEM 450, FEI, Hillsboro, OR, USA). All the samples were coated with gold before observation. The reflection spectra were measured by HITACHI U-4100 spectrophotometer (HITACHI, Tokyo, Japan) at the scan speed of 300 nm/min with the slit width of 8.00 nm and an integral sphere. FT-IR spectra of the samples were obtained on a Nicolet Avatar 320 spectrometer (KBr pellets, Nicolet Instrument Corp., Madison, WI, USA). X-ray photoelectron spectra (XPS) were measured on a ESCALAB250 multifunction surface analysis system (Thermo Fisher Scientific Corp., Boston, MA, USA) using Al-Ka radiation. UV-Vis absorbance spectra were obtained by JASCO UV-550 (JASCO Corp., Tokyo, Japan). Thermogravimetric analysis (TG) was conducted using a Switzerland Mettler-Toledo TGA/SDTA851 thermal analyzer (Mettler-Toledo Corp., Switzerland) from 30 °C to 800 °C with a heating rate of 10 °C/min under nitrogen atmosphere.

### 3.3. Synthesis of Aniline 2,5-Double Sulfonic Acid Diazonium Salt

In a typical process, 2.75 g (0.01 mol) of aniline-2,5-disulfonic acid monosodium salt was dissolved in 30 mL of water by the addition of stoichiometric sodium carbonate to adjust the pH of the solution to 7. Then, 0.76 g (0.011 mol) of sodium nitrite was dissolved in the above solution; the mixture was named as solution A. Meanwhile, 3.5 mL of concentrated hydrochloric acid (12 mol/L) was diluted with 26.5 mL of water in a 150 mL beaker and named as solution B. Solution A was then poured into solution B under moderate magnetic stirring in an ice-bath. After 6 h of stirring, a certain amount of sulfamic acid was added to decompose the excess sodium nitrite until the potassium iodide-starch test paper did not turn blue. Finally, the diazonium salt was stored in an ice-bath for further use.

### 3.4. Surface-Modification of MWCNTs

**Scheme 1:**
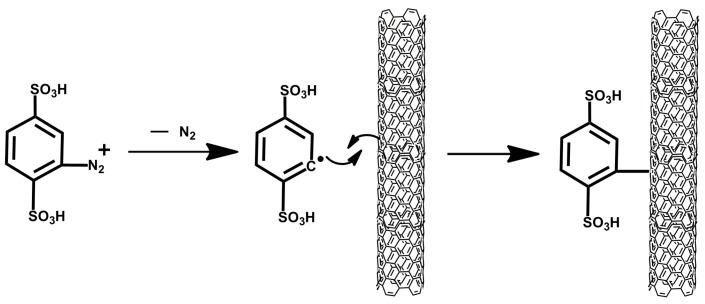
Schematic showing the hydrophilic modification process of MWCNTs.

For surface modification (Scheme 1), 1.0 g of MWCNTs was added in 50 mL of water and ultrasonically mixed for 30 min. Then, 60 mL of the aniline 2,5-double sulfonic acid diazonium salt solution was added dropwise to the mixture. The whole process (~3 h) was carried out under ultrasonication at 300 W in a 55 °C water bath. The mixture was stirred for 6 h after the complete addition of diazonium salt. Finally, the mixture was washed three times with water in centrifugation/redispersion cycles to remove the impurities completely. The purified product was ultrasonically redispersed in water by adjusting the pH to 7~8 to obtain 0.1 wt % of MWCNTs dispersion for further using.

### 3.5. Preparation of Monodisperse PS Emulsion

Monodisperse PS colloidal spheres with different particle sizes were prepared by a non-soap emulsion polymerization method reported in our previous article [33]. Typically, PS emulsion with hydrodynamic particle size of 261 nm was synthesized as follows: 0.075 g of SDS was pre-mixed with 135 mL of deionized water for 15 min in a 250 mL four-necked flask equipped with a nitrogen flow tube, a mechanical stirrer, a thermometer, and a reflux condenser. Then, 15 g of styrene monomer was added and stirred for another 15 min at 250 r/min in 85 °C water bath and N_2_ flow. Then, 0.15 g of KPS was added to the reaction system and the polymerization was carried out at 85 °C for 6 h under N_2_ flow. Finally, PS emulsion with 10 wt % theoretical solid content was obtained and stored for further use. PS particle size was controlled through the dosage of SDS. Table 1 shows the properties of three kinds of PS spheres.

### 3.6. Fabrication of Photonic Crystal Films

**Scheme 2:**
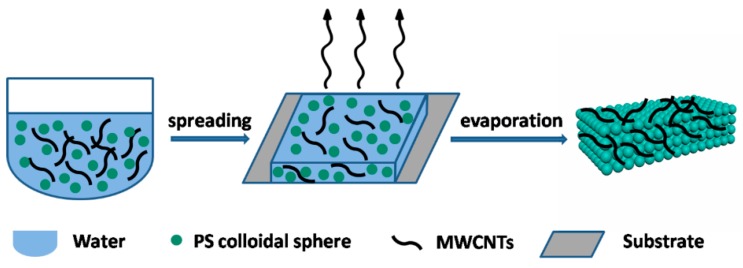
Schematic showing the thermal assisted self-assembly process.

As shown in Scheme 2, thermal-assisted self-assembly method was applied to fabricate photonic crystal films. Glass slides (25 mm × 75 mm) were used as assembling substrate. The pre-prepared PS emulsion (10 wt % in solid content) was mixed with different amounts of modified MWCNTs by sufficient ultrasonication to form homogeneous PS/MWCNTs emulsion mixtures. *n*-Propanol (5% *v*/*v*) was premixed with the emulsion mixture to increase spreadability on the substrate surface. Then, the emulsion mixture was spread dropwise onto the substrate, which was placed on a heating panel at 80 °C. Finally, with the evaporation of water, PS colloidal spheres together with MWCNTs self-assembled to form photonic crystal films. Maintaining a stable surrounding environment in the whole assembly process is important.

## 4. Conclusions

In conclusion, we successfully modified crude MWCNTs through radical addition reaction by using aniline 2,5-double sulfonic acid diazonium salt. The modified MWCNTs possess a hydrophilic surface and good water dispersity, which are suitable for solution processing. For the first time, the MWCNTs dispersion was blended with PS emulsion to fabricate photonic crystals. The structural color visibility was considerably enhanced because of the light-adsorption ability of MWCNTs. In addition, the mechanical strength of the films was improved because of the crosslinking effect between MWCNTs and PS particles. The utilization of MWCNTs to build composite photonic crystals with in improved properties is significant for their potential applications, such as in paints and displays.

## Figures and Tables

**Table 1:** Synthesis parameters and properties of three kinds of PS spheres.

Sample	SDS (g)	Size (nm)		PDI ^3^	Zeta Potential (mV)
		Da ^1^ Dh ^2^			
(**a**)	0.050	278	317	0.009	−35.1
(**b**)	0.075	239	261	0.017	−31.7
(**c**)	0.100	198	235	0.013	−38.3

^1^ Da: statistical average diameter through SEM; ^2^ Dh: hydrodynamic size of the particles measured by DLS; ^3^ PDI: particle size distribution index.

## Supplementary Materials

The following are available online at http://www.mdpi.com/1420-3049/21/5/547/s1, Figure S1: Photograph of dispersion behavior of MWCNTs: (**a**) crude MWCNTs; (**b**) modified MWCNTs; (**c**) homogeneous mixtures of PS emulsion doped with modified MWCNTs, Figure S2: UV-Vis spectra of the upper, middle, and bottom of the MWCNTs dispersion, Figure S3: (**a**) XPS spectra of crude and modified MWCNTs; (**b**) High-resolution XPS spectrum of S_2p_core in modified MWCNTs, Figure S4: TGA weight loss curves of crude and modified MWCNTs, Figure S5: SEM image of pure PS film with hydrodynamic particle size of 317 nm.
