# Supplementary material for: Hydrophilic Modification of Multi-Walled Carbon Nanotube for Building Photonic Crystals with Enhanced Color Visibility and Mechanical Strength
Source: Molecules. 2016 Apr 28;21(5):547. doi: 10.3390/molecules21050547 (PMC6273566; doi:10.3390/molecules21050547)
Supplement: Supplementary file 1 [file molecules-21-00547-s001.pdf]

Supplementary Materials: Hydrophilic Modification of Multi-Walled Carbon Nanotube for Building Photonic Crystals with Enhanced Color Visibility and Mechanical Strength

Feihu Li, Bingtao Tang, Jinghai Xiu and Shufen Zhang

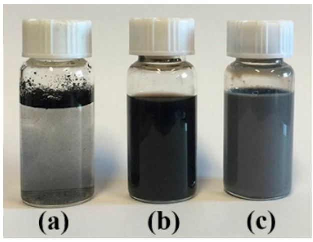

Figure S1. Photographs of dispersion behavior of MWCNTs: (**a**) crude MWCNTs; (**b**) modified MWCNTs; (**c**) homogeneous mixtures of PS emulsion doped with modified MWCNTs.

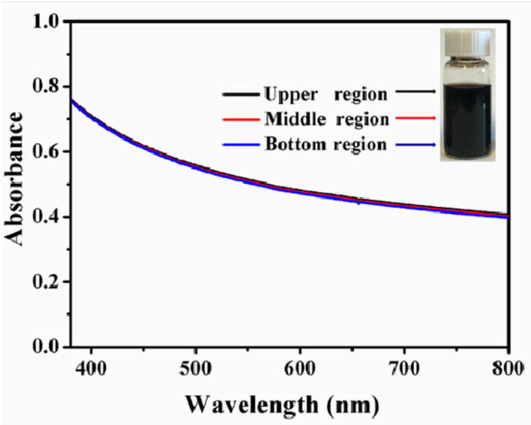

Figure S2. UV–Vis spectra of the upper, middle, and bottom of the MWCNTs dispersion.

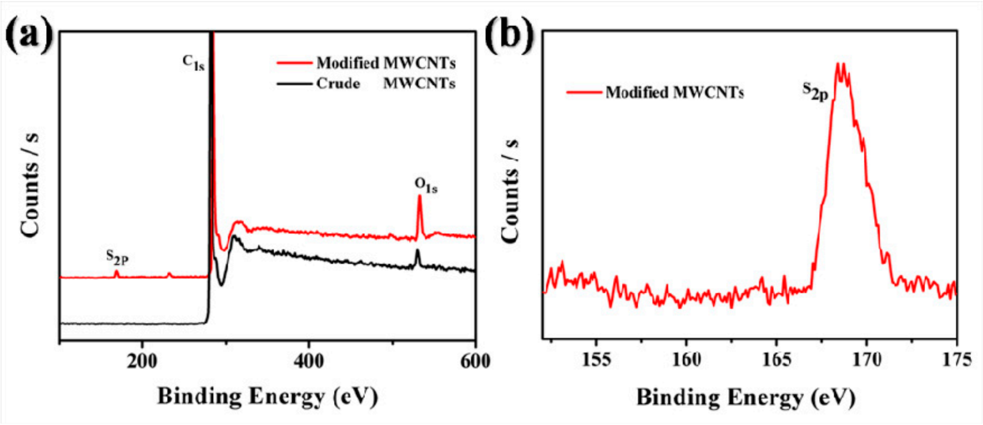

Figure S3. (**a**) XPS spectra of crude and modified MWCNTs; (**b**) High-resolution XPS spectrum of S_{2p}core in modified MWCNTs.

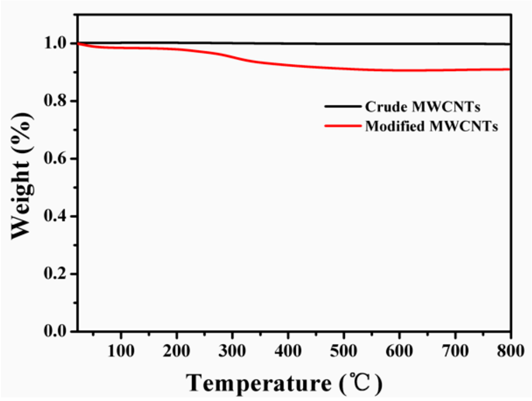

Figure S4. TGA weight loss curves of crude and modified MWCNTs.

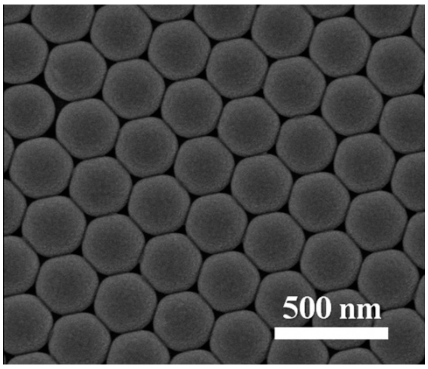

Figure S5. SEM image of pure PS film with hydrodynamic particle size of 317 nm.
